# Ethephon-Mediated Bloom Delay in Peach Is Associated With Alterations in Reactive Oxygen Species, Antioxidants, and Carbohydrate Metabolism During Dormancy

**DOI:** 10.3389/fpls.2021.765357

**Published:** 2021-10-14

**Authors:** Md Tabibul Islam, Jianyang Liu, Sherif M. Sherif

**Affiliations:** Virginia Tech, School of Plant and Environmental Sciences, Alson H. Smith Jr. Agricultural Research and Extension Center, Winchester, VA, United States

**Keywords:** peach, ethephon, hydrogen peroxide, antioxidants, soluble sugars, starch, bloom delay

## Abstract

Ethephon (ET) is an ethylene-based plant growth regulator (PGR) that has demonstrated greater efficacy in delaying bloom in deciduous fruit species. However, the underlying mechanisms by which ET modulates dormancy and flowering time remain obscure. This study aimed to delineate the ET-mediated modulations of reactive oxygen species (ROS), antioxidants, and carbohydrate metabolism in relation to chilling and heat requirements of “Redhaven” peach trees during dormancy. Peach trees were treated with ethephon (500ppm) in the fall (at 50% leaf fall), and floral buds were collected at regular intervals of chilling hours (CH) and growing degree hours (GDH). In the control trees, hydrogen peroxide (H_2_O_2_) levels peaked at the endodormancy release and declined thereafter; a pattern that has been ascertained in other deciduous fruit trees. However, H_2_O_2_ levels were higher and sustained for a more extended period than control in the ET-treated trees. ET also increased the activity of ROS generating (e.g., NADPH-oxidase; superoxide dismutase) and scavenging (e.g., catalase, CAT; glutathione peroxidase) enzymes during endodormancy. However, CAT activity dropped significantly just before the bud burst in the ET-treated trees. In addition, ET affected the accumulation profiles of starch and soluble sugars (hexose and sucrose); significantly reducing the sucrose and glucose levels and increasing starch levels during endodormancy. However, our study concluded that variations in ROS levels and antioxidation pathways, rather than carbohydrate metabolism, could explain the differences in bloom time between ET-treated and -untreated trees. The present study also revealed several important bud dormancy controlling factors that are subject to modulation by ethephon. These factors can serve as potential targets for developing PGRs to manipulate bloom dates in stone fruits to avoid the ever-increasing threat of spring frosts.

## Introduction

Peach (*Prunus persica*) is a highly prized seasonal fruit worldwide. The production of peach in several fruit-growing regions in the United States and worldwide often suffers from damages caused by late-spring frosts. In the United States, frost damage can lead to economic losses more than any other weather-related phenomenon (Wisniewski et al., 2016). The risk of frost damage is projected to rise due to global climate change, and more variable, extreme weather events. At the forefront of this challenge is the cultivated fruits species. Unlike their wild counterparts, cultivated species are excluded from the path of natural evolution through practices of asexual reproduction (e.g., grafting), thereby becoming unable to adapt to rapid climatic changes. Global warming has the potential to reduce winter chill, threatening the production of fruit and nut crops in several regions (Luedeling et al., 2011). Another remarkable effect of climate warming is the changes in plant phenology, which has caused many woody species to suffer increased frost damages in the US and Europe (Augspurger, 2013; Ma et al., 2018). In fact, over the last three decades, apple blooming in Europe has advanced with an average of 2–3days per decade (Vitasse et al., 2011, 2018; Hoffmann and Rath, 2013), and such advance of blooming is projected to be 0.5–2.1days per decade throughout the rest of this century (Unterberger et al., 2018). A similar rate was documented in peach, which significantly increased its vulnerability to frost damage (Ma et al., 2018). Therefore, there has been a need for a chemical treatment (s) that could delay budburst and bloom date of vulnerable cultivars and species beyond the last day of frost (Nzokou and Nikiema, 2008; Grijalva-Contreras et al., 2011; Liu and Sherif, 2019a; Liu et al., 2021).

Bud dormancy is an adaptive mechanism of deciduous fruit trees to cope with the severe cold during the winter and is crucial for tree phenology, especially flowering. According Lang (1987), dormancy can be divided into three types: paradormancy, endodormancy, and ecodormancy. In paradormancy, the meristem growth is inhibited by signals from other plant organs, e.g., the inhibition of axillary buds by the shoot apex in what is generally known as apical dominance. In this form of dormancy, the growth of lateral buds can be resumed once the apex is removed. Unlike paradormancy, growth arrest during endodormancy is primarily regulated by endogenous signals from within the meristem itself. In ecodormancy, growth inhibition is caused by external factors like temperature and day length. Endodormant buds require a period of chilling to release from dormancy. This chilling period is referred to as chilling requirements (CR) and can be calculated according to several models and represented as chilling hours (CH), chilling units (CU), or chilling portions (CP; Weinberger, 1950; Richardson et al., 1975). CR is genotype-dependent and highly diverse across species and varieties. Some peach varieties require as high as 1,000 CH and others require only 50 CH (Fadón et al., 2020). During ecodormancy, buds need a period of warm temperatures in the range of 4.5–25°C, known as heat requirements (HR), to initiate the subsequent developmental phases of flowering and fruit set. Heat requirements, often expressed as growing degree hours (GDH), are also a genotype-dependent trait (Guo et al., 2014). In peach, bloom time is determined, at least partially, by the genotype’s CR and HR (Layne and Bassi, 2008).

Ethephon (2-chloroethylphosphonic acid; ET) is a plant growth regulator (PGR) known to delay the bloom in peach and other stone fruits, such as sweet cherry and apricot (Durner and Gianfagna, 1991). Extensive studies have reported that fall-applied ET delays bloom date in different stone fruits species by a few days to more than 2weeks, depending on the concentration and application timing (Liu and Sherif, 2019b). Indeed, in a recent study, we have shown that ET (500ppm) applied at 50% leaf fall increased the cold hardiness, CR, and HR of peach flower buds and delayed the bloom time for 7days (Liu et al., 2021). Though ET appears to be a promising candidate in delaying bloom and avoiding spring frosts, its use is often associated with several detrimental effects, such as gummosis, terminal dieback, and yield reduction (Funt and Ferree, 1986; Crisosto et al., 1987; Krewer et al., 2005; Liu et al., 2021). The long interval between ET application and the bloom delay response also adds to the uncertainty of its efficacy as a frost avoidance tool. However, ET-mediated bloom delay can serve as an effective model to elucidate the biochemical and molecular mechanisms underlying bud dormancy and bloom time in peach, which could eventually lead to effective and practical spring frost mitigation approaches.

Bud cells have the capacity to produce and scavenge different forms of ROS at various levels, as a result of a balance between their formation and detoxification, with a tight link to dormancy maintenance and release (Beauvieux et al., 2018; Hernández et al., 2021a). The ROS generation, including H_2_O_2_, may work as major signaling molecules for the endodormancy release (Pérez et al., 2021). It has also been reported that exogenous H_2_O_2_ may substitute for chilling requirements in Japanese pear (Kuroda et al., 2005). The enhanced H_2_O_2_ level has also been reported in sweet cherry and peach in response to hydrogen cyanamide, a dormancy-breaking chemical (Pérez et al., 2008; Ionescu et al., 2017). The unsaturation reaction that converts linoleic acid (C18:2) to linolenic acid (C18:3) could also generate ROS (i.e., H_2_O_2_) as an indirect product. Such elevated H_2_O_2_ levels could also inactivate the fatty acid desaturase enzymes (Norman et al., 1991). Fatty acids unsaturation plays a crucial role in peach dormancy maintenance and release (Erez et al., 1997). Therefore, the balance between ROS production and scavenging is crucial for efficient fatty acid unsaturation and subsequently dormancy release and bud break. Indeed, higher levels of glutathione and ascorbate, the key scavengers of ROS, were found to be closely associated with the dormancy release in deciduous species, including peach and almond (Siller-Cepeda et al., 1992; Guillamón et al., 2020). The upregulation of the oxidation-reduction process during the endodormancy release and bud break has also been demonstrated in many other deciduous species (Beauvieux et al., 2018; Yu et al., 2020). However, reports on the temporal changes of the ROS and associated antioxidants/scavengers during dormancy are scarce, especially in relation to chill and heat accumulation.

Carbohydrate metabolism also plays a crucial role in dormancy and bloom regulation. Reserved carbohydrates serve as a primary energy source for bud development during dormancy. Also, chilling-induced soluble sugar accumulation enhances bud cold tolerance during dormancy by decreasing the freezing point of free water, thus preventing the intracellular ice crystal formation in the buds during dormancy (Zwieniecki et al., 2015; Tixier et al., 2019). However, there has been discrepancy about the accumulation kinetics of various forms of carbohydrates during dormancy cycle, which could be due to differences in the CR and length of the dormancy period among the investigated cultivars. For instance, in the low-chill peach cultivar “Okinawa,” the glucose level remains unchanged during the endodormancy and increases significantly during the ecodormancy stage (Hernández et al., 2021b). In contrast, in a high-chill peach cultivar., “Yumyeong,” glucose level declines throughout the endodormancy and increases in ecodormancy, and similar accumulation pattern was observed in the fructose content (Hernández et al., 2021a). It has been also reported that starch accumulates gradually during endodormancy and hydrolyzes into soluble sugars by amylase at dormancy release and bud break (Gholizadeh et al., 2017; Kaufman and Blanke, 2017), whereas others show starch degradation throughout the dormancy cycle (Zhang et al., 2018).

In the present study, changes in the accumulation pattern of various forms of carbohydrates, e.g., glucose, fructose, sucrose, and starch, and their associated genes expression were investigated in peach cultivar “Red haven” to elucidate the role of carbohydrate metabolism in bloom time regulation in peach. The ET-mediated bloom delay model was also used to study the dynamics of ROS, antioxidation pathways, and their associated genes throughout the dormancy. The characterization of various biochemical, enzymatic activity, and the genes expression was further interpreted in relation to the chilling and heat accumulation, providing insights into the mechanism of ET-mediated regulations of bud dormancy and flowering in peach.

## Materials and Methods

### Plant Materials, Treatments, and Experimental Design

This study was conducted at the Alson H. Smith Jr. Agricultural Research and Extension Center (AREC), Winchester, VA, United States (39°06ʹ36.0 ʹN 78°16ʹ48.0 ʹW). The “Redhaven” peach trees grafted to “Lovell” rootstock were planted in 2012. Treated and control trees were arranged in the orchard according to the Randomized Complete Block Design (RCBD), with three orchard rows being used as blocks. In each block, two groups, each with two adjacent trees, were randomly assigned to receive ethephon treatments at 500ppm (Liu et al., 2021) or untreated control, and two buffer trees were left between treated and untreated trees to avoid spray drift. Motivate^®^ (Fine American Inc., Walnut Creek, CA, United States) containing 21% of ethephon (2-chloroethylphosphonic acid) was used in this study. Ethephon (500ppm) was mixed with a nonionic surfactant Regulaid (250ppm, Kalo Inc., Overland Park, KS, United States) and sprayed with an air-blast sprayer at 50% leaf fall (October 24, 2019). Chilling and heat accumulations were recorded from meteorological data obtained from onsite temperature data loggers (EasyLog, Lascar, Erie, PA, United States). Chilling accumulation was calculated as chilling hours (CH; according to 0–7.2°C model, Weinberger, 1950). Heat accumulation was recorded as GDH (based on the 4.5–25°C model, Richardson et al., 1975). The CR for dormancy release was determined when 50% of bud break was achieved under forcing conditions according to the methods described by Liu et al. (2021). Floral buds were sampled in 15ml tubes at 200 CH, 400 CH, 600 CH, 800 CH, and 1,000 CH; 1,000 GDH and 3,000 GDH, immediately frozen in liquid nitrogen and stored at−80°C for later biochemical and gene expression analyses. Bloom progression was evaluated according to a method previously described by Liu et al., 2021. The Bloom rate was calculated as the ratio of open blossoms to the initial number of buds per tree, and the flowering date (F_50_) for each treatment was determined when the blooming rate reached 50%.

### Reactive Oxygen Species Quantification

To quantify ROS, including superoxide anion radical (O_2_^•−^) and hydrogen peroxide (H_2_O_2_), 100mg of fresh ground floral bud tissues was extracted using 50mm potassium phosphate buffer (pH 7.0). The content of O_2_^•−^ was determined by the hydroxylamine oxidation method (Lee et al., 2009). Briefly, a mixture of 100μl extracts and 25μl of hydroxylamine hydrochloride (10mm) was incubated at 25°C for 1h, then reacted with 100μl of 1% of sulfanilamide and 100μl of 7mm α-naphthylamine solution at 25°C for 20min. The absorbance was recorded at 530nm with a Synergy H1 hybrid microplate reader (BioTek, Oakville, ON, Canada). The O_2_^•−^ content was obtained using a linear calibration curve of NaNO_2_. For H_2_O_2_ assay, 500μl of the extract was mixed with 500μl of 0.1% titanium chloride in 20% (v/v) H_2_SO_4_ and centrifuged at 10000rpm for 5min. The absorbance was immediately read at 410nm using the above-mentioned microplate reader. H_2_O_2_ concentration was calculated using the coefficient of absorbance 0.28μm^−1^cm^−1^ as described previously (Lee et al., 2009).

### Glutathione Redox Assay

Approximately 100mg fresh ground bud tissues were homogenized in 5% of 5-sulfosalicylic acid and centrifuged at 12,000rpm for 10min at 4°C for the extraction of the glutathione. The oxidized and reduced glutathione content were determined by microplate assay using the GSH/GSSG Kit GT40 (Oxford Biomedical Research Inc., Rochester Hills, MI, United States) according to the manufacturer’s instructions.

### Assay of Antioxidative Enzymes Activity

To quantify the antioxidant enzymes, 100mg of fresh ground tissues was homogenized in 50mm potassium phosphate buffer (pH-7.0) and centrifuged at 12000rpm for 10min. The supernatant was transferred to clean 2ml tubes and used for further analyses.

The enzyme activity of NADPH oxidase was assayed using the methods outlined by Jiang and Zhang (2002) with minor modification. Briefly, the NADPH-dependent O_2_ generating activity was determined by monitoring the reduction of sodium 3,3ʹ-{[(phenylamino)carbonyl] -3,4-tetrazolium}-bis (4-methoxy-6-nitro) benzene-sulfonic acid hydrate (XTT; Sigma-Aldrich) by O_2_^•−^ to determine the NADPH-oxidase activity. Rates of O_2_^−^ generation were calculated using an extinction coefficient of 2.16×10^4^M^−1^cm^−1^. The NADPH-oxidase activity was expressed as nmol of O_2_^−^ min-^1^ mg^−1^ of protein.

The activity of superoxide dismutase (SOD), catalase (CAT), and glutathione peroxidase (GPx) enzymes was assayed using assay kits from BioVision Inc. (Milpitas, CA, United States) according to the manufacturer’s instructions. The SOD activity was expressed as inhibition rate (%) per mg of protein. One unit of CAT was expressed as the amount of CAT that decomposed 1.0nmol of H_2_O_2_ min^−1^. The amount of GPx that causes the decrease of 1.0nmol of NADPH min^−1^ was expressed as one unit.

### Soluble Sugar and Starch Analyses

Soluble sugars were extracted using 80% ethanol according to a method described previously by La et al., 2019. The glucose, sucrose, and fructose contents were determined using the Megazyme Sucrose/D-Fructose/D-Glucose Assay Kit (Megazyme, Bray, Ireland) according to the manufacturer’s protocol. Total soluble sugar was determined by summing the content values of glucose, fructose, and sucrose in each sample.

Starch content was determined using the method described by Lee et al. (2020) with some modifications. After sugar extraction, the pellet was dried, suspended with 1ml of distilled water, and heated at 80°C for 10min. The pH of the supernatant was adjusted to 5.1 by adding 400μl of 200mm acetate buffer. Starch was digested by adding 100μl of the reaction mixture (0.2U of amyloglucosidase and 40U of α-amylase) to the solution and then incubating at 37°C for 24h. After centrifugation at 14,000rpm for 5min, the glucose in the supernatant was measured using the Megazyme Sucrose/D-Fructose/D-Glucose Assay Kit (Megazyme, Bray, Ireland) according to the manufacturer’s protocol. Starch concentration was estimated as 0.9×glucose concentration.

### Isolation of Total RNA and Gene Expression Analyses

For the gene expression analysis, total RNA from bud samples was extracted according to a CTAB method described previously by Sherif et al., 2016, with some modifications. Briefly, around 200mg ground tissue of each sample was homogenized with CTAB extraction buffer [2% (w/v) CTAB (cetyl trimethyl ammonium bromide), 2% (w/v) PVP (polyvinylpyrrolidone, mol wt 40,000), 100mm Tris-HCl (pH 8.0), 25mm EDTA, 2M NaCl, 0.05% spermidine trihydrochloride, and 2% of β-mercaptoethanol] and incubated at 65°C for 30min. After incubation, samples were centrifuged at 13,200rpm for 15min at room temperature. The supernatant was treated with an equal volume of chloroform-isoamyl alcohol (24:1; Sigma-Aldrich), then centrifugated at 13,200rpm for 15min at room temperature. The aqueous supernatant was transferred to a 2ml tube and 1/4 volume of 10M LiCl was added to the supernatant. Tubes were mixed well by inverting them several times and then incubated at −20°C for 2h. The total RNA was pelleted by centrifugation at 14,000rpm for 30min at 4°C. The supernatant was discarded and the pellet was washed with 0.5ml of 80% ethanol and centrifuged again at 14000rpm for 5min at 4°C. The supernatant was removed and the pellet was dried at room temperature. The RNA pellet was dissolved in RNase free water and the RNA quantity was estimated using a Synergy H1 hybrid reader (BioTek, Oakville, ON, Canada). All RNA samples were treated with DNase (EZ BioResearch, St Louis, Missouri, United States) and then purified using the EZ RNA Clean-Up Plus DNase Kit (EZ BioResearch). The cDNA was synthesized from 2μg of DNase-treated RNA using a cDNA synthesis kit (Applied Biosystems, Foster City, United States) and according to the manufacturer’s instructions. Quantitative real-time PCR (qRT-PCR) was performed using CFX connect real-time detection system (Bio-Rad, Mississauga, ON, Canada), SsoFast^™^ EvaGreenR Supermix (Bio-Rad), and gene-specific primers (Supplementary Table S1). The gene expression of target genes was first normalized to the expression of two peach house-keeping genes, *β-actin* and *Ubiquitin*. The accumulation of gene transcripts in each sample was calculated according to the 2^−ΔΔ^Ct method (Livak and Schmittgen, 2001) and relative to the expression in control trees at 20 CH. All the qRT-PCR reactions were performed in three biological and three technical replicates, and results were analyzed using the gene study function of the CFX manager software (Bio-Rad).

### Statistical and Multivariate Analyses

The statistical analyses of the data were conducted using the software SAS 9.1.3 (SAS Institute Inc., Cary, NC, United States). Tukey’s HSD test was employed to compare the means of separate replicates. Statistical significance was postulated at *p* < 0.05. The student t-test was conducted between control and the ET treatment at each time point, and the asterisks (^*^, ^**^, and ^***^) in the figures indicate significant differences (*p* < 0.05,<0.01, and<0.001, respectively). The multivariate analysis, including the partial least squares-discriminant analysis (PLS-DA), was performed using MetaboAnalyst 5.0.^1^

## Results

### Effects of the Ethephon on Chilling and Heat Requirements, Dormancy Release, and Blooming Time

**Figure 1:**
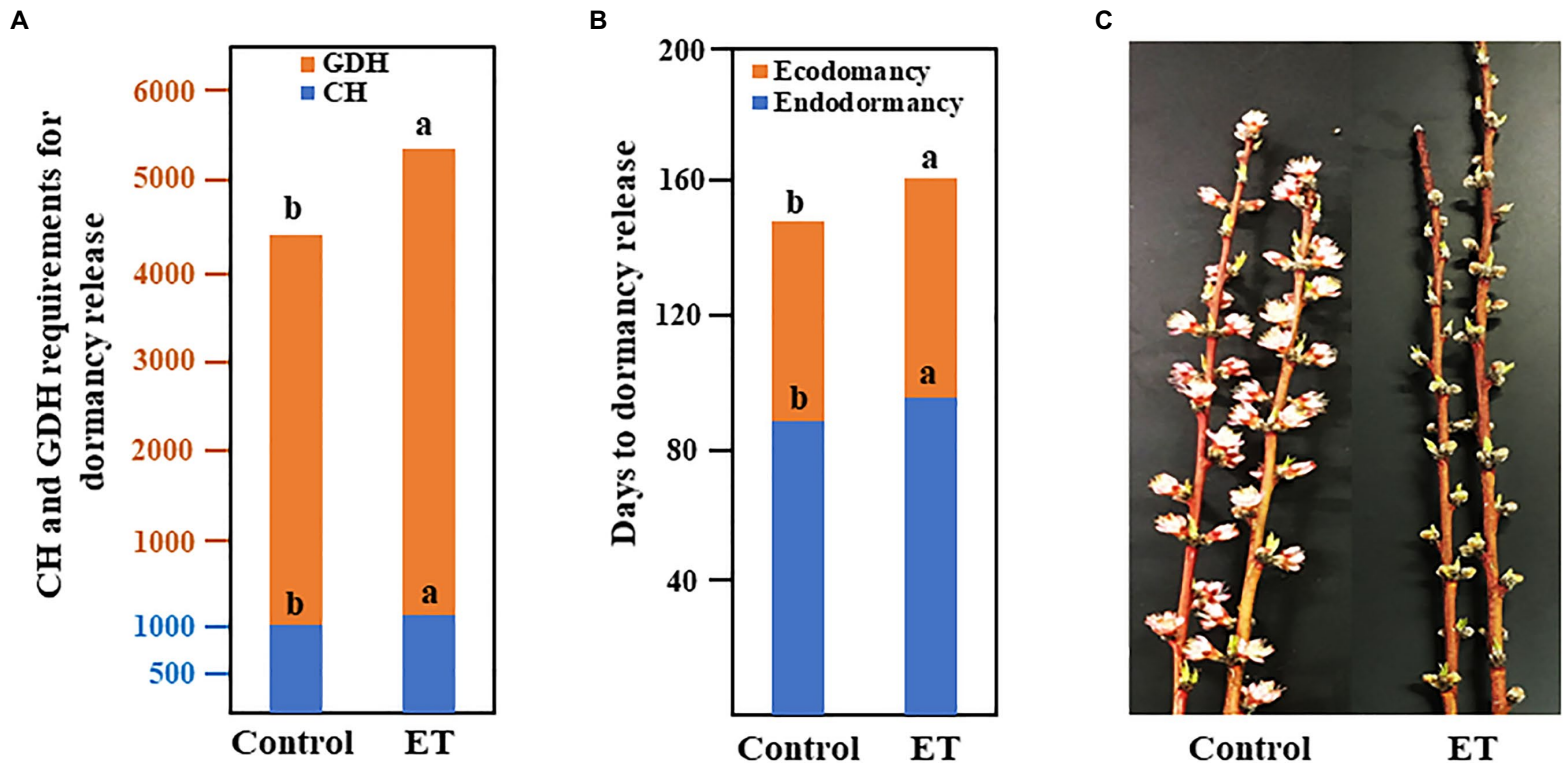
Chilling and heat requirements **(A)**, duration of the endo- and ecodormancy **(B)**, and bloom delaying effects **(C)** of ethephon (ET) in peach “Red haven.” Data represent the mean of the three independent replicates. Bars labeled with different letters are significantly different at *p* < 0.05, according to Tukey’s HSD test.

Floral buds had no signs of flushing under forcing conditions on September 30 and hence, these date were considered the endodormancy initiation date. The CH accumulation was first recorded on October 7, 2019. Chilling requirements (CR) for the control trees were satisfied at 1046 CH, whereas CR for ethephon (ET)-treated trees was 131 CH higher (*p* < 0.05) than control (Figure 1A). Heat requirements (HR) were recorded after fulfillments of the CR and the accumulation of GDH was first observed on January 23, 2020. Similar to the CR, ethephon significantly increased the heat requirements and ET-treated trees required 901 GDH (*p* < 0.05) more than control to achieve 50% bloom (Figure 1A). Fall-applied ET at 500ppm significantly extended the duration of both endodormancy (ED) and ecodormancy (EC; Figure 1B), resulting in a delay in flowering date by 6days (Figure 1C). The full description of ethephon effects on CR, HR, cold hardiness, flowering time, fruit set, and tree health in peach was previously published (Liu et al., 2021).

### Ethephon Mediated Alteration in ROS Accumulation Pattern and Glutathione Redox Balance

**Figure 2:**
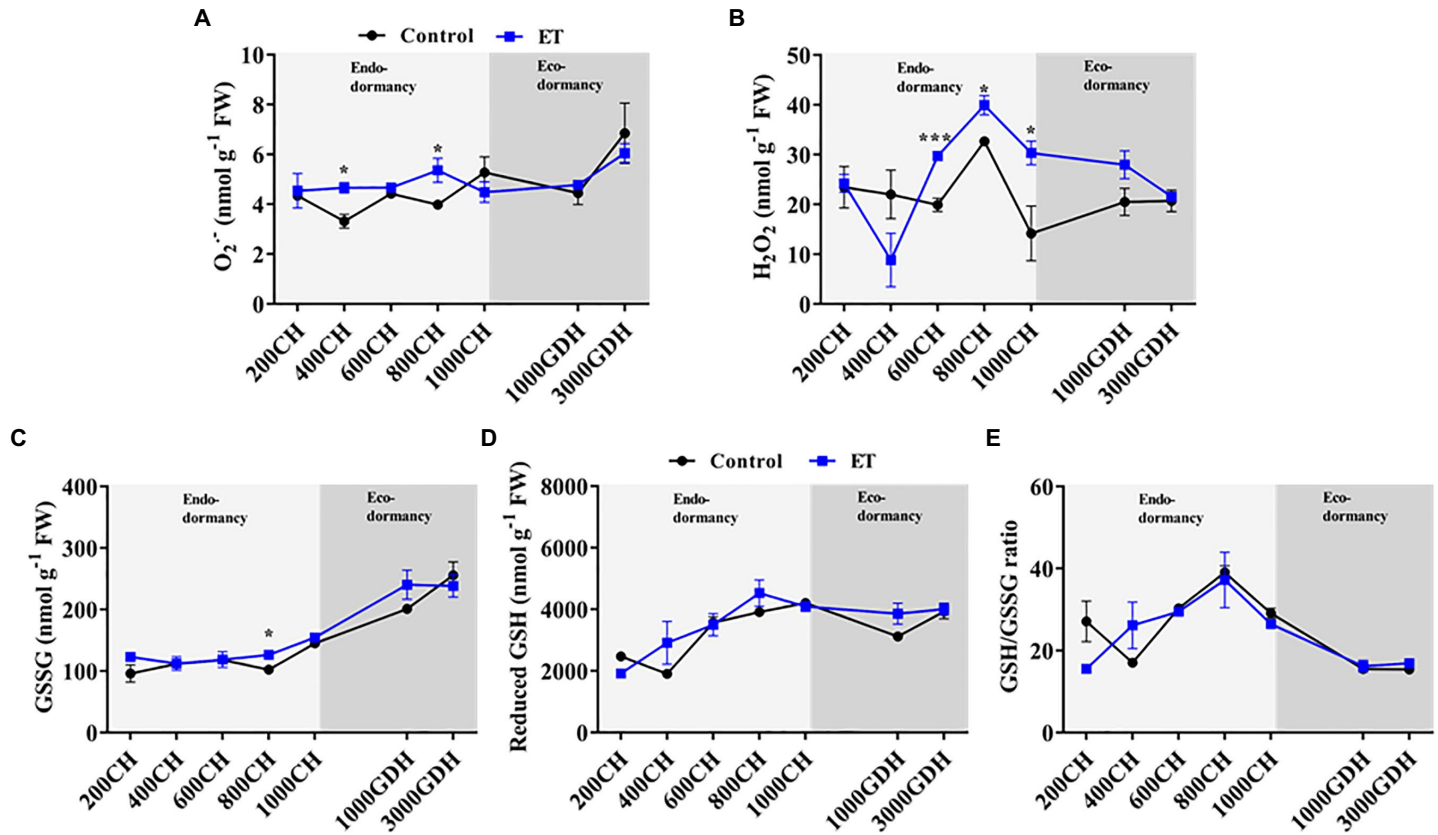
Effects of the ethephon (ET) on the temporal changes of the reactive oxygen species (ROS) and glutathione-based redox during the dormancy in peach “Red haven” flowering buds. Superoxide radical (O_2_^•−^; **A)**, hydrogen peroxide (H_2_O_2_; **B)**, oxidized glutathione (GSSG; **C)**, reduced glutathione (GSH; **D)**, and the GSH/GSSG ratio **(E)**. Data represent the mean±SEM for *n*=3. Asterisks indicate significant differences between the control and ET-treated plant buds at different chilling hours (CH) and growing degree hours (GDH); ^∗^*p* < 0.05; and ^∗∗∗^*p* < 0.001.

In order to determine the temporal changes of ROS and their alterations by the ET treatment, we quantified the content of the superoxide radical (O_2_^•−^) and hydrogen peroxide (H_2_O_2_), in the floral buds at different stages of dormancy. The data showed that overall O_2_^•−^ content in both ET-treated and untreated trees remained unchanged during chill accumulation but was significantly higher in the ET-treated buds at 400 CH and 800 CH (Figure 2A). At the end of ecodormancy (at 3000 GDH), O_2_^•−^ content sharply increased compared to the early ecodormancy stage (at 1000 GDH) irrespective of the treatment. The H_2_O_2_ level remained unchanged at 200 CH to 600 CH then peaked at the 800 CH in control plants (Figure 2B). ET treatment, on the other hand, increased H_2_O_2_ levels in the dormant flower buds and that increase was significantly higher than control at 600 CH (*p* < 0.001; 49.3%), 800 CH (*p* < 0.05; 22.2%), and 1,000 CH (*p* < 0.05;113.7%). No significant differences were observed between the control and ET-treated trees during the ecodormancy stage (Figure 2B), but in general H_2_O_2_ content showed downward and upward trends in ET and control plants, respectively, as trees moved from endodormancy toward bud break.

The levels of oxidized glutathione (GSSG) in the control and ET-treated plants both remained around 100nmolg^−1^ during the endodormancy and gradually increased to 250nmolg^−1^ at 3000 GDH during ecodormancy (Figure 2C). In contrast, the reduced form of glutathione (GSH) accumulated gradually during endodormancy (Figure 2D), and peaked at the endodormancy release (800–1,000 CH) and slightly decreased during ecodormancy with no significant differences between control and the ET treatment (Figure 2D). The resulted glutathione redox balance (GSH/GSSG) in both the control and ET treatment showed similar patterns of the GSH, peaking at 800CH, then steadily declining until budbreak (Figure 2E).

### Effects of Ethephon on Activity of the Antioxidative Enzymes and Their Associated Genes

**Figure 3:**
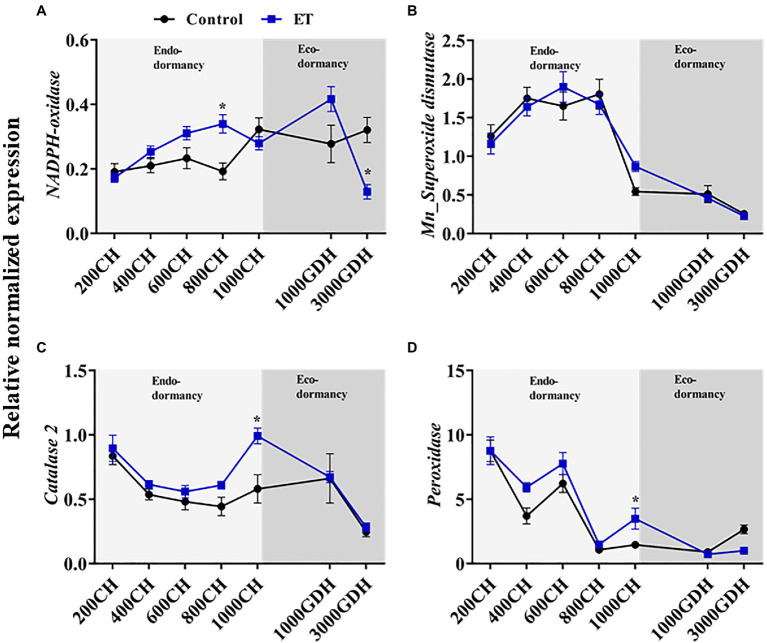
Ethephon (ET)-mediated changes of the antioxidative-related genes expression in peach “Red haven” flowering buds during the dormancy. *NADPH-oxidase* **(A)**, *Mn_superoxide dismutase* (*Mn_SOD*; **B)**, *catalase 2* (*CAT2*; **C)**, and *glutathione peroxidase* (*Gpx*; **D)**. Data represent the mean±SEM for *n*=3. Asterisks indicate significant differences between the control and ET-treated plant buds at different chilling hours (CH) and GDH; ∗*p* < 0.05.

**Figure 4:**
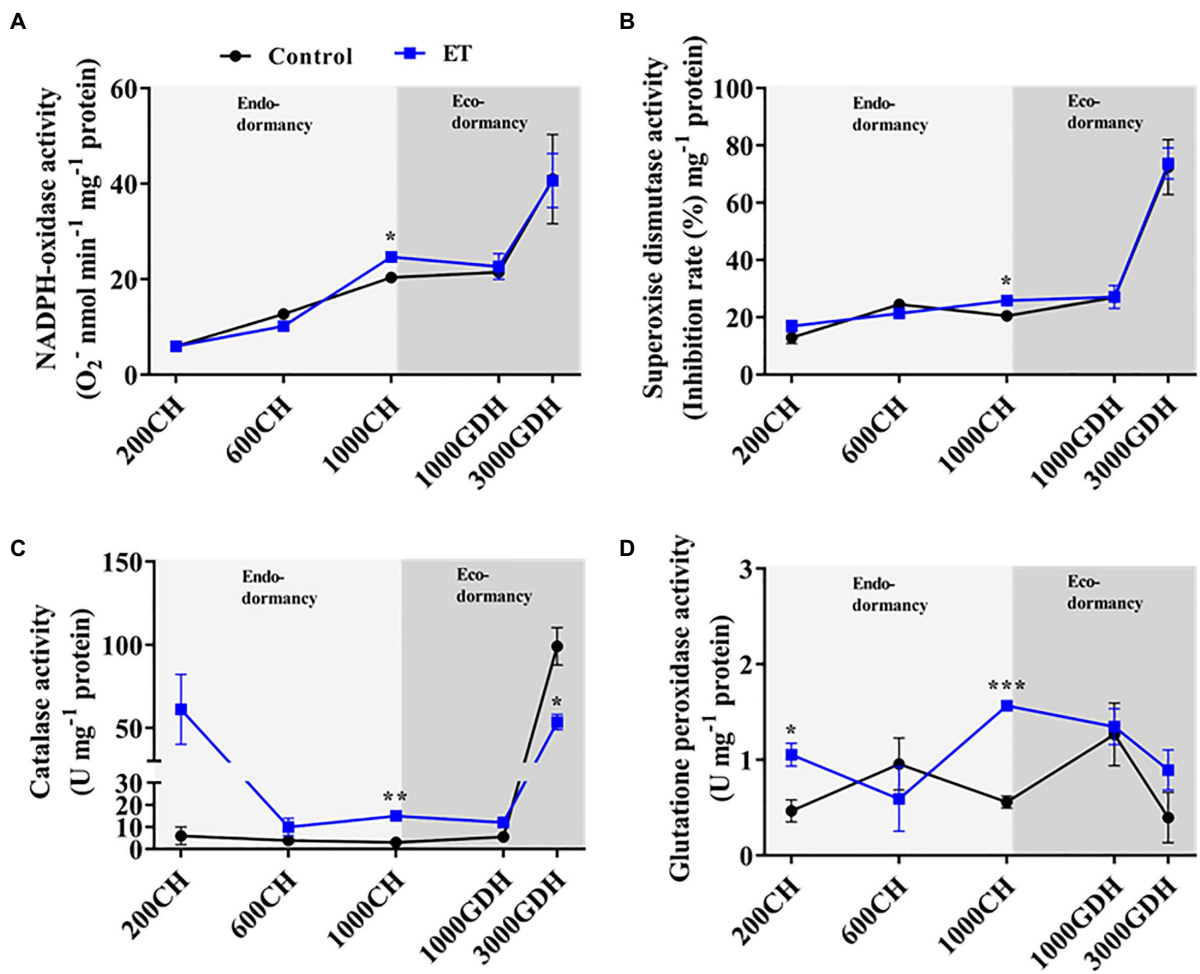
Changes in the activity of the antioxidative enzymes in peach “Red haven” floral buds during the dormancy in responses to ethephon (ET) treatment. NADPH-oxidase activity **(A)**, superoxide dismutase activity **(B)**, catalase activity **(C)**, and glutathione peroxidase activity **(D)**. Data represent the mean±SEM for *n*=3. Asterisks indicate significant differences between the control and ET-treated plant buds at different chilling hours (CH) and GDH; ^∗^*p* < 0.05; ^∗∗^*p* < 0.01; and ^∗∗∗^*p* < 0.001.

To further evaluate the effects of ethephon on the ROS formation/scavenging balance during dormancy, the enzymatic activity of NADPH oxidase, SOD, CAT, and GPx was evaluated in ET-treated and untreated trees. NADPH oxidase and SOD activity showed a similar pattern throughout the dormancy cycle (Figures 3A,B), with a slight increase during the endodormancy and a sharp increase by the end of ecodormancy. The only significant difference (*p* < 0.05) between control and ET-treated buds was observed at 1000 CH (Figures 4A,B). CAT and the GPx activities had a similar trend during endodormancy, and the ET treatment enhanced both the enzyme activity at 200 CH (10.2- and 2.3 folds, respectively) and1000 CH (4.8- and 2.8-fold, respectively) compared to control (Figures 3C,D). Additionally, the CAT activity sharply increased at the end of ecodormancy (3,000 GDH) in both control ET treatment, and the increase rate was significantly lower in ET treatment (*p* < 0.05).

At the transcriptional level, *NADPH-oxidase* and *MnSOD* gene expression levels tended to increase as the endodormancy progressed (Figures 4A,B). Apparently, the ET treatment increased *NADPH-oxidase* expression gradually during the endodormancy and that increases were significantly higher (*p* < 0.05; 76.2%) than control at 800 CH (Figure 4A). On the other hand, the expression of *CAT2* showed gradual decline at 200 CH through 600 CH then peaked at the end of endodormancy in both control and ET-treated plants; however, ET-treated trees showed a significant increase in *CAT2* transcript levels at 1000 CH (*p* < 0.05; 70.7%) compared to control (Figure 4C). Similar to *CAT2*, the expression of *peroxidase* showed a decline as the dormancy progressed, with a slight, yet significant, increase recorded in the ET-treated buds at1000 CH (Figure 3D).

### Effects of Ethephon on the Gene’s Expression Involved in Lipid Metabolism

**Figure 5:**
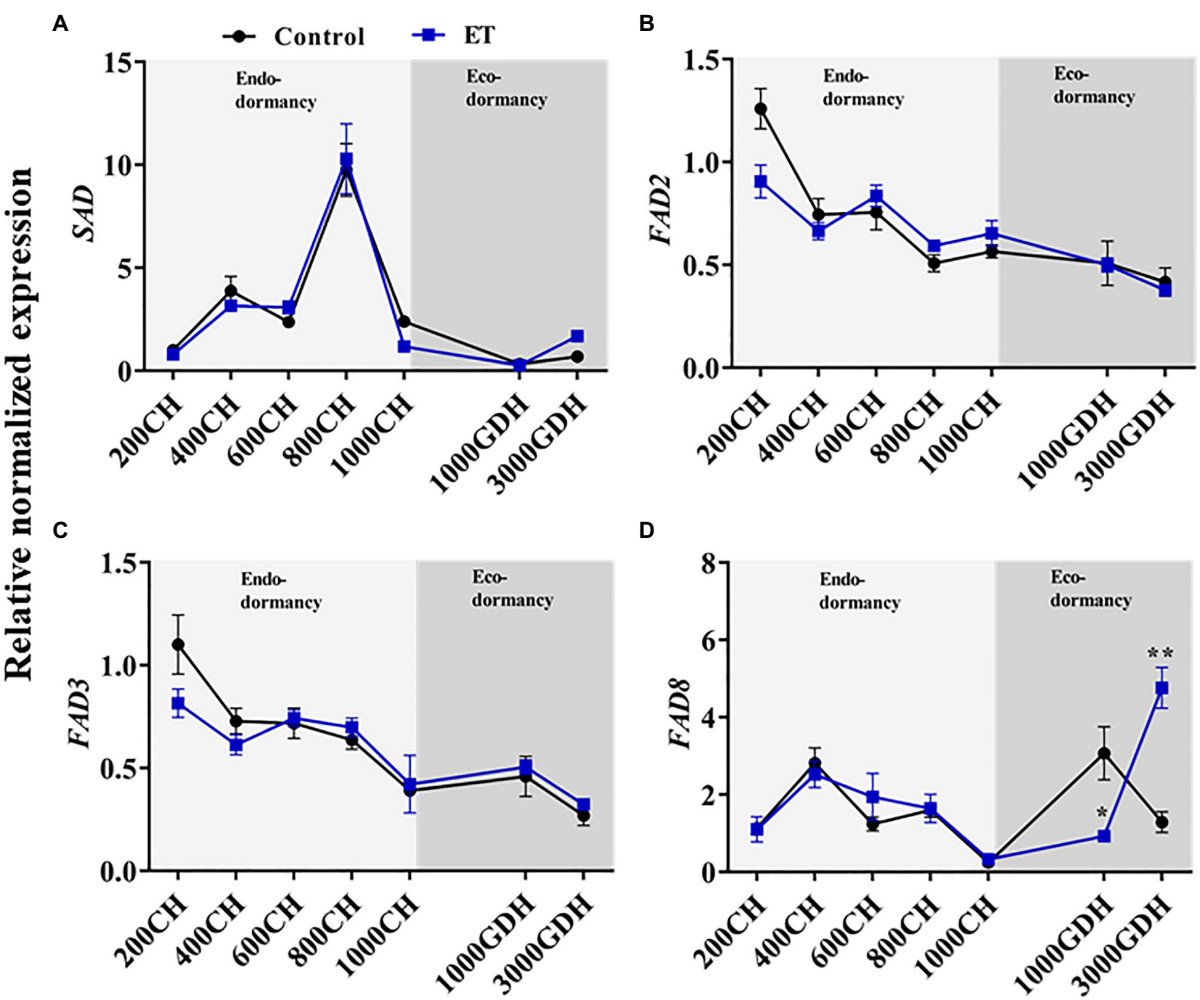
Alterations of the fatty acid metabolism-related genes during the dormancy by the ethephon (ET) treatment in peach “Red haven” floral buds. *Stearoyl-acyl carrier protein desaturase* (*SAD;* **A)**, *fatty acid desaturase 2* (*FAD2*, **B)**, *fatty acid desaturase 3* (*FAD3*, **C)**, and *fatty acid desaturase 8* (*FAD8*, **D)**. Data represent the mean±SEM for *n*=3. Asterisks indicate significant differences between the control and ET-treated plant buds at different chilling hours (CH) and GDH; ^∗^*p* < 0.05; and ^∗∗^*p* < 0.01.

In order to evaluate the changes in the lipid metabolic process during the dormancy in relation to changes in ROS levels, we assessed the gene expressions of *stearoyl-acyl carrier protein desaturase* (*SAD*), *fatty acid desaturase 2* (*FAD2*), *fatty acid desaturase 3* (*FAD3*), and *fatty acid desaturase 8* (*FAD8*) in control and ET-treated buds. The *SAD* expression patterns were highly similar between the control and ET treatment, with both peaking at 800 CH and sharply declining at 1000 CH, maintaining a similar expression level over the rest of the dormancy period (Figures 5A). *FAD2* and *FAD3* transcript levels showed a gradual decrease as the dormancy progressed, but no significant differences were observed between treatments at any point throughout the dormancy cycle (Figures 5B,C). The expression level of the *FAD8* was also similar between control and ET-treated buds during endodormancy but showed significant differences between treatment at the ecodormancy stage. At the early stage of ecodormancy, the *FAD8* gene expression was significantly lower in ET-treated buds (*p* < 0.05; −69.8%) but showed significantly higher (*p* < 0.01; 268.7%) levels at the end of ecodormancy compared to control (Figure 5D).

### Modulations of the Carbohydrate Metabolism by Ethephon

**Figure 6:**
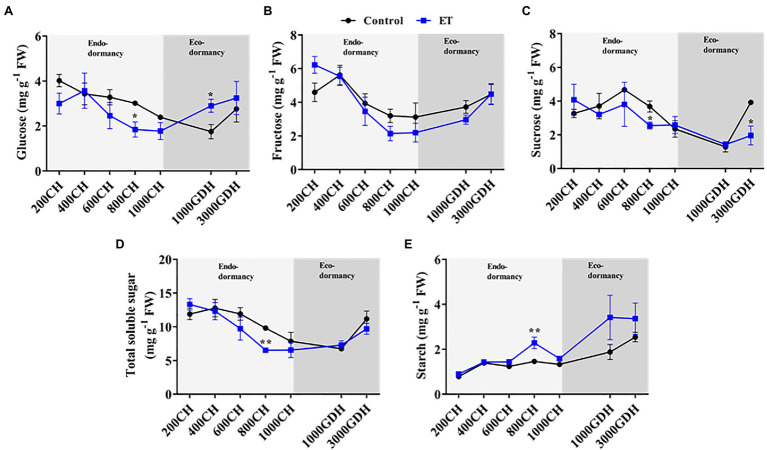
Effects of the ethephon (ET) on the temporal changes of the soluble sugar and starch accumulation during the dormancy in peach “Red haven” floral buds. Glucose **(A)**, fructose **(B)**, sucrose **(C)**, total soluble sugar **(D)**, and starch **(E)**. Data represent the mean±SEM for *n*=3. Asterisks indicate significant differences between the control and ET-treated plant buds at different chilling hours (CH) and GDH; ^∗^*p* < 0.05; and ^∗∗^*p* < 0.01.

Peach bud’s content of soluble sugars (glucose, fructose, and sucrose) and starch was analyzed in ET-treated and untreated buds at different levels of CR and HR. Overall, in both control and ET-treated buds, the hexose (glucose and fructose) levels gradually decreased during endodormancy and increased during ecodormancy (Figures 6A,B). ET treatment significantly reduced the glucose level at 800 CH (*p* < 0.05) compared to control (Figure 6A). The glucose tended to increase as the buds proceeded toward ecodormancy, with significantly higher levels observed in the ET-treated buds (*p* < 0.01). Similarly, the sucrose level declined as endodormancy progressed and was significantly lower in the ET-treated buds at 800 CH. A slight increase in sucrose level was observed by the time of bud break (3,000 GHD), but again that increase was significantly lower (*p* < 0.05; 49.9%) in the ET-treated buds (Figure 6C). Unsurprisingly, the total soluble sugar accumulation pattern during dormancy followed the same patterns of hexose and sucrose (Figure 6D). Unlike soluble sugars, the starch level was slightly increasing as the dormancy progressed and it was generally higher in the ET-treated buds, with a significant increase (*p* < 0.01; 56.4%) in the ET-treated trees observed at 800CH (Figure 6E).

**Figure 7:**
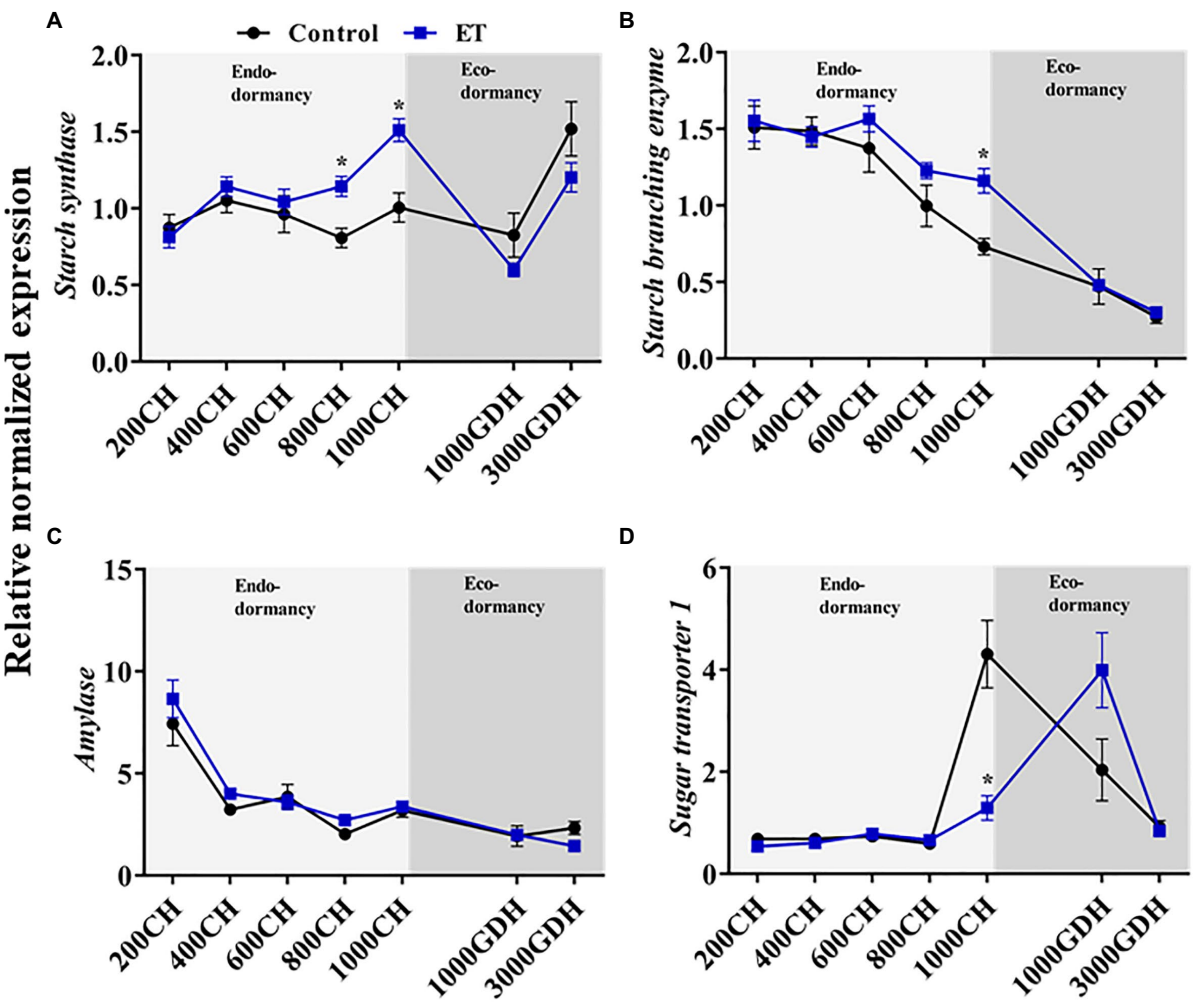
Ethephon (ET)-mediated changes of the starch synthesis and degradations and the sugar transportation-related genes expression in peach “Red haven” floral buds during the dormancy. *Starch synthase* **(A)**, *starch branching enzymes* **(B)**, *amylase* **(C)**, and *sugar transporter 1* **(D)**. Data represent the mean±SEM for *n*=3. Asterisks indicate significant differences between the control and ET-treated plant buds at different chilling hours (CH) and GDH; ^∗^*p* < 0.05.

We further analyzed the expression of the *starch synthase* (*SS*), *starch branching enzyme* (*SBE*), and *amylase* (*AMY*) to investigate the changes in the starch synthesis and degradation processes during dormancy (Figure 7). Transcripts of *SS* showed significantly higher levels in the ET-treated plants compared to control during endodormancy, particularly at 800 CH (*p* < 0.05; 41.6%) and 1,000 CH (*p* < 0.05; 50.3%; Figure 7A). Unlike *SS*, a sharp decline of *SBE* gene expression was observed in both control and ET-treated buds during both stages of dormancy, but *SBE* transcripts levels were significantly higher (*p* < 0.05) in ET-treated trees at 1000 CH (Figure 7B). We also analyzed the expression *AMY* which is involved in the starch degradation process. *AMY* expression was relatively high at the early stage of endodormancy (200 CH), then declined thereafter, with no significant differences being observed between treatments (Figure 7C). The gene expression of the sugar transporter (*sugar transporter 1*; *STP1*) increased significantly in the control plants at the end of endodormancy (at 1000 CH). The peak in *STP1* transcript levels occurred later (1,000 GDH) in the ET-treated buds, but such increase was not significant when compared to the control (Figure 7D).

### Partial Least Squares-Discriminant Analysis

**Figure 8:**
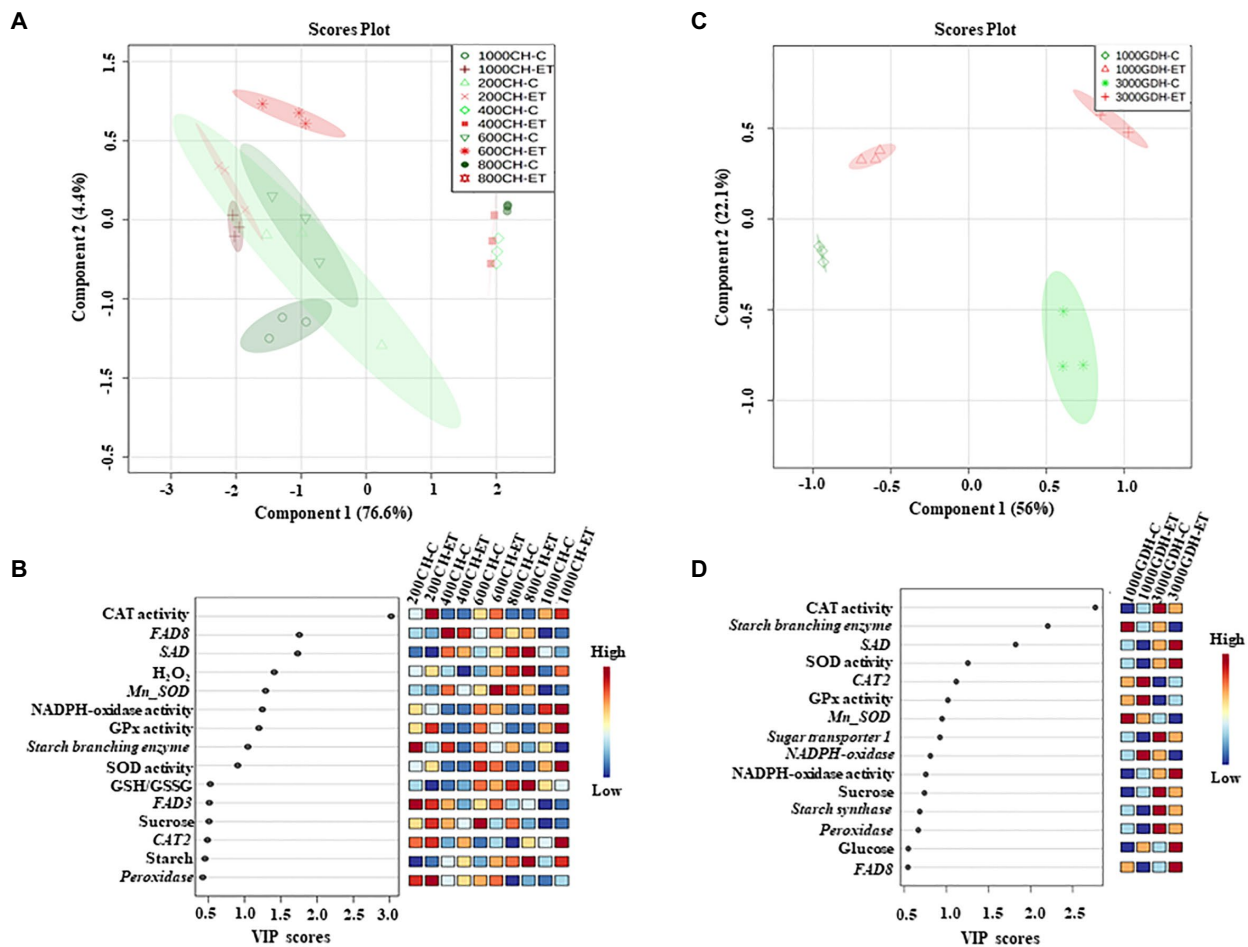
2D score plot of partial least squares-discriminate analysis (PLS-DA) during endodormancy **(A)** and ecodormancy **(B)**. Significant factors that contributed to component 2 with variable importance in projection (VIP≥0.5) measure in PLS-DA during endodormancy **(C)** and ecodormancy **(D)**. The color boxes on the right indicate the relative concentrations of each variable under this study. C: control; ET: ethephon; CH: chilling hours; GDH; growing degree hours; and VIP: variable importance in projection.

The partial least squares-discriminant analysis (PLS-DA) was performed to identify the significant factors among the tested ROS and carbohydrate metabolism components that could contribute to the ET effects in delaying bloom. Overall, the PLS-DA score plot for the endodormancy showed that component 1 which represents chilling hours (CH) could explain 76.6% of the total variation (Figure 8A). However, component 2 which represents the treatments could explain only 4.4%. The variables that contributed substantially (variable importance in projection, VIP scores ≥1.0) to component 2 were H_2_O_2_, antioxidative enzymes (NADP oxidase, SOD, CAT, and GPx activity), and fatty acids synthesis-related genes (FAD8 and SAD; Figure 8B).

Similar to endodormancy, at ecodormancy, the component 1 primarily represents the two different stages of ecodormancy (1,000 GDH and 3,000 GDH) and could predict 56% of the variations, whereas component 2 that could explain 22.1% of data variations represents the treatments (Figure 8C). The antioxidative enzymes (CAT, SOD, and GPX) were the major variables (VIP scores ≥1.0) that contributed to component 2 (Figure 8D).

## Discussion

Peach buds are susceptible to late-spring frosts. Extending the dormancy period and delaying bloom has been considered a promising frost avoidance strategy. Over the last couple of years, we have demonstrated that fall-applied ethephon (ET) extends the dormancy period and delays bloom in peach (Liu et al., 2021). However, the associated adverse effects of ET limit its commercial application (Funt and Ferree, 1986; Crisosto et al., 1987; Krewer et al., 2005; Liu and Sherif, 2019a). Understanding the ET-mediated modulation of diverse metabolic processes, including ROS, antioxidants, and carbohydrates during dormancy, could lead to feasible alternative treatment (s) for delaying bloom and preventing the spring frost-related damages in stone fruits.

Previous reports showed that exogenous H_2_O_2_ can substitute for the chilling requirement in Japanese Pear (Kuroda et al., 2005) and that H_2_O_2_ increase during dormancy may be required for the breaking of bud dormancy in grapevine (Pérez et al., 2008). Interestingly, Kuroda et al., 2005 found that an exogenous application of H_2_O_2_ (2.5%) to the endodormant buds of Japanese pear increased bud break, but it required a higher concentration of H_2_O_2_ (10%) to attain a similar bud break rate when the application was made during ecodormancy. Treatments with hydrogen cyanamide (HC), a dormancy-breaking agent, were also found to substitute chilling requirements in grapevine and Japanese pear by inducing H_2_O_2_ accumulation in the buds (Kuroda and Sugiura, 2002; Liang et al., 2019). In both species, the accumulation profile of H_2_O_2_ during endodormancy was similar to what we observed in the present study (Figure 2B), where H_2_O_2_ reaches its peak at endodormancy release and declines during ecodormancy (Kuroda and Sugiura, 2002; Pérez et al., 2021). Such pattern was also observed with the ET treatment, except that ET maintained H_2_O_2_ at higher levels for an extended period compared to control. Maintaining elevated H_2_O_2_ levels for a longer time could explain the previously reported ET effects on extending the dormancy period and delaying bloom in peach (Liu et al., 2021). In fact, it was reported that bloom delay in grapevine could be achieved by treating the buds with aminotriazole, an irreversible inhibitor of H_2_O_2_ detoxifying enzyme CAT, which consequently sustain a high level of H_2_O_2_ for an extended period (Pérez et al., 2021). In addition to H_2_O_2_, rapid accumulation of the O_2_^•−^ was also detected in grapevine buds in response to HC application (Sudawan et al., 2016). In the present study, we showed that chilling accumulation had minimal effects on the O_2_^•−^ level. Instead, O_2_^•−^ accumulation was induced at the end of ecodormancy in response to heat accumulation regardless of the treatment (Figure 2A), suggesting that O_2_^•−^ induction might be associated with bud burst, rather than endodormancy release.

The levels of H_2_O_2_ during dormancy are maintained by a balance between its production and scavenging. NADPH oxidase is a membrane-bound enzyme catalyzing the production of O_2_^•−^-, which can be further converted to H_2_O_2_ by SOD (Van Gestelen et al., 1997). In this study, ET induced the NADPH-oxidase and SOD’s gene expression and enzymatic activity during endodormancy (Figures 3, 4). The CAT and the GPx are the major enzymes that detoxify the lethal H_2_O_2_ (Islam et al., 2019)_._ The activity of both enzymes as well as their corresponding transcript levels was significantly higher in ET-treated buds at the end of endodormancy. The CAT activity could explain the downward trend of the H_2_O_2_ from the end of endodormancy to ecodormancy, where an upward pattern was observed in the control at 3000 GDH (Figure 2B).

H_2_O_2_ can be produced as a byproduct of the fatty acid unsaturation (Wang and Faust, 1990; Gabay et al., 2019), but it can also inactivate fatty acid desaturases (Norman et al., 1991). Fatty acid unsaturation plays a central role in dormancy release (Norman et al., 1991; Erez et al., 1997; Gabay et al., 2019). For example, inhibiting the unsaturation of fatty acids, especially the conversion of linoleic acid (C18:2) to α-linolenic acid (C18:3), delayed bud dormancy release in peach (Gibson et al., 2004). Also, the exogenous application of α-linolenic acid was found to promote dormancy break in Japanese pear (Sakamoto et al., 2010). The ROS detoxification system is crucial for fatty acid unsaturation during dormancy (Norman et al., 1991, Erez et al., 1997, Gabay et al., 2019). The elevated levels of non-enzymatic antioxidants, e.g., glutathione and ascorbate, are closely associated with the endodormancy release in deciduous species, including apple, peach, and almond (Wang and Faust, 1990; Siller-Cepeda et al., 1992; Guillamón et al., 2020). In the present study, it was observed that ET treatment did not alter the GSH metabolism (Figures 2C–E) but maintained high H_2_O_2_ levels for an extended period during endodormancy which could have a negative effect on fatty acid unsaturation. Indeed, ET altered the gene expression of *fatty acid desaturase 8* (*FAD8*) during ecodormancy (Figure 5). FAD8 desaturates linoleic acid to α-linolenic acid in the plastid (Dar et al., 2017), suggesting that ET may affect dormancy and bloom in peach through interfering with redox hemostasis, which in turn suppresses the expression of *FAD8* and the subsequent α-linolenic acid production.

Carbohydrates are essential for bud growth regulation during dormancy and also play a crucial role in cold tolerance (Anderson et al., 2010). Soluble sugars are believed to act as compatible solutes in dormant tissues to confer tolerance to cold and desiccation stresses (Lloret et al., 2018). Variations in the accumulation dynamics of the different forms of carbohydrates have been associated with contrasting chilling requirements or different bud types (Gholizadeh et al., 2017; Fadón et al., 2018; Hernández et al., 2021a,b). For example, distinct hexose (glucose and fructose) accumulation patterns were found during dormancy in peach cultivars with contrasting chilling requirements, with glucose declining during dormancy in the high-chill cultivar “Yumyeong,” but remaining unchanged in the low-chill peach cultivar “Okinawa” (Hernández et al., 2021a,b). We also found a similar declining trend of hexose and sucrose as chill accumulation progressed, with a higher decreasing rate of the hexose and sucrose observed in the ET treatment during the endodormancy than control (Figure 6). Moreover, starch showed high levels at the end of endodormancy, especially in the ET-treated buds, which was supported by the enhanced expressions of a starch synthase gene compared to the control (Figures 6, 7).

Chilling-induced enhancement of amylolytic activity promotes cold tolerance of grapevine and walnut buds by increasing the starch hydrolysis and subsequently increasing the soluble sugar level, thus reducing the free water availability in the buds (Sauter, 1988; Ben Mohamed et al., 2010; Gholizadeh et al., 2017). On the other hand, in the sweet cherry floral primordium, starch accumulated following the same pattern as chilling accumulation (Fadón et al., 2018). We also found the increasing trend of starch during endodormancy, which was significantly higher in ET treatment (Figure 6E). The discrepancy between these results could be due to the cellular differences between the buds. For instance, the buds of grapevine and walnut are mixed/compound buds, which comprise both vegetative and floral primordia (Vasconcelos et al., 2009), whereas peach buds are either vegetative or floral (Penso et al., 2020). Unlike vegetative buds, which contain highly vacuolated mature cells, floral buds mainly consist of ovary primordial cells that are dense with cytoplasm, lacking in vacuoles, and low in water availability (Yooyongwech et al., 2008). Given these differences, our finding suggests in floral buds, starch, rather than soluble sugars may be essential in coping with the potential freezing stress during dormancy.

Besides its role in cold tolerance, carbohydrate metabolism is also evidently involved in the transition from endodormancy to ecodormancy (Beauvieux et al., 2018). During ecodormancy, a gradual accumulation of the hexose and starch has been reported in different deciduous fruits tree species, including peach (Gholizadeh et al., 2017; Hernández et al., 2021a,b). In this study, significant induction of glucose at the early stage of ecodormancy (at 1000 GDH) was found in the ET treatment compared to the control (Figure 6A). It is likely that the glucose induction at the early stage of ecodormancy contributed to the delay of dormancy release and flowering. This may result from improper glycolysis limiting the acetyl CoA availability and hindering the fatty acid synthesis, which is crucial for the cell membrane formation, thus delaying bud break (Cernac and Benning, 2004).

## Conclusion

Over the past two decades, there has been growing evidence that sugars and ROS production during dormancy are tightly connected to chilling accumulation and dormancy release; yet, it remained unclear how redox hemostasis and carbohydrate metabolism during dormancy could explain the ethephon-mediated bloom delay in peach and other fruit tree species. The present study confirmed that H_2_O_2_ accumulates proportionally with the increment in chill hours, reaching its peak at the endodormancy release. We also showed that ethephon altered H_2_O_2_ accumulation during endodormancy, sustaining a high level of H_2_O_2_ for 200 CH beyond the control’s peak. H_2_O_2_ induction during endodormancy was likely supported by NADPH-oxidase and SOD enzymes. Ethephon application not only increased the activity of these enzymes but also stimulated the enzymatic antioxidation pathways, such as those mediated by CAT and GPx, which is necessary for maintaining ROS at sub-lethal levels. This study also provided a plausible link between ethephon, ROS, fatty acid unsaturation, and bloom delay in peach that undoubtedly deserves more investigations in the future. Our results also revealed a unique relationship between starch and TSS in peach buds during dormancy, with the former being more dominant during endodormancy. Overall, the biochemical and molecular analyses in the present study revealed that the fall-applied ET can modulate the forms and levels of ROS, antioxidants, and carbohydrates during peach bud dormancy. However, the PLS-DA discrimination analysis showed that CAT activity was the primary variable alerted by the ET during both the endo- and ecodormancy, suggesting that the modulation of ROS and antioxidation pathways, rather than carbohydrate metabolism, could be the primary factors responsible for the ET-mediated bloom delay in peach.

## Data Availability Statement

The original contributions presented in the study are included in the article/Supplementary Material, and further inquiries can be directed to the corresponding author.

## Author Contributions

MI performed the majority of the experiments, analyzed the data, and wrote the manuscript. JL assisted with sample collection, tissue preparation, and editing the manuscript. SS conceived and designed the experiments, edited the manuscript, and oversaw the study. All authors have read and approved the final manuscript.

## Funding

This project was partially funded by the Virginia Department of Agriculture and Consumer Services (418953) and the Virginia’s Agricultural Council (449846).

## Conflict of Interest

The authors declare that the research was conducted in the absence of any commercial or financial relationships that could be construed as a potential conflict of interest.

## Publisher’s Note

All claims expressed in this article are solely those of the authors and do not necessarily represent those of their affiliated organizations, or those of the publisher, the editors and the reviewers. Any product that may be evaluated in this article, or claim that may be made by its manufacturer, is not guaranteed or endorsed by the publisher.

## Footnotes

^1^ https://www.metaboanalyst.ca/MetaboAna-lyst/home.xhtml
