# Supplementary material for: Ethephon-Mediated Bloom Delay in Peach Is Associated With Alterations in Reactive Oxygen Species, Antioxidants, and Carbohydrate Metabolism During Dormancy
Source: Front Plant Sci. 2021 Oct 14;12:765357. doi: 10.3389/fpls.2021.765357 (PMC8551920; doi:10.3389/fpls.2021.765357)
Supplement: Supplementary file 1 [file Data_Sheet_1.pdf]

Supplementary Table S1. Specific primers used for qRT-PCR.

Gene	Description	Gene Bank Accession Number	Forward and reverse sequence (5'-3')
Mn_SOD	Mn_Superoxide dismutase	Prupe.6G042300.1	CCCCACCATCGTCAAATTAC<br>TCTCGAACAGGGGTCAGATT
CAT2	Catalase 2	Prupe.5G011400.1	GGAAACAATTTCCCCGTCTT<br>TCTCTTGGATGTGCGACTTG
NADPH-oxidase	NADPH-oxidase	Prupe.5G138300.1	ATGTCTTGGCCCTTCATTTG<br>ATGGAGACACAGCAGCACAC
SS	Starch synthase 3	Prupe.4G020100.1	GCGCACTTTATTGGAAAAGC<br>GTGGAGCAATTGCAGGATTT
SBE	Starch branching enzymes 2	Prupe.1G354000.1	AGGGATAGCGCTTCACAAGA<br>CAGGATGGCCAAACTCATTT
AMY	Amylase	Prupe.5G125700.1	AGGGAGGCTGAGACCATTTT<br>TGACGGATGAGACTGAGTGC
STP1	Sugar transporter 1	Prupe.4G037800.1	CCCAAAAGGAATTGGGATCT<br>TGGAACTAACCACCCCAAAG
POX	Peroxidase	Prupe.3G115300.1	TCGCAATGCTATGAGTCAGG<br>TCGCAACCATTTACAAAGCA
SAD	Stearoyl-[acyl-carrier-protein] 9-desaturase 6	Prupe.8G018700.1	CACCACCGCCATCTCTAAC<br>ACTCCCCTTTGGGTAGTGC
FAD2	Fatty acid desaturase 2	Prupe.6G278800	TTCGAGTTTTTCGGTCGAG<br>CTTCGGAATGGCTGCTTTA
FAD3	Fatty acid desaturase 3	Prupe.7G261900	TTTCAAAAGCCCCATGGTA<br>CAATAGGCTGCCAAGCTGT
FAD8	Fatty acid desaturase 8	Prupe.6G056100	GAGGCCAAGCTGATTTTGA<br>GGCCAAACATACCAAGTGC
ACT	Actin	Prupe.6G163400.1	TTGTGCTGGACTCTGGTGA<br>GCAAGGTCCAGACGAAGAA
UBQ	Ubiquitin	Prupe.3G004600.1	GTCTTCCGACACCATCGAC<br>CCTGCGAAGATGAGACTGT

All primers were designed directly from sequences that available in the public database.

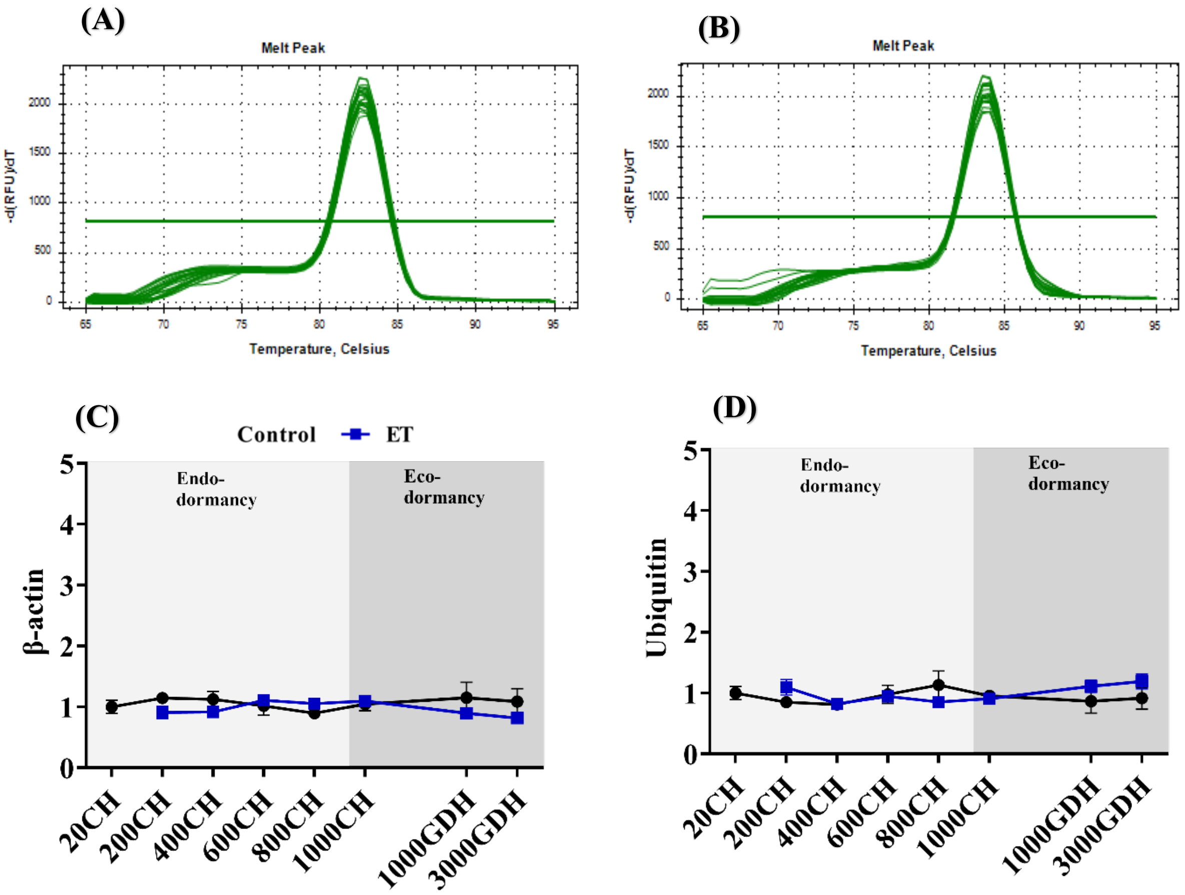

Supplementary Figure 1 | The transcriptional stability of two housekeeping genes. Melt curve plot of *β-actin* (A) and *Ubiquitin* (B). The expression of two genes encoding *β-actin* (C) and *Ubiquitin* (D) in control and ethephon (ET) treated buds at different chilling hours (CH) and growing degree hours (GDH). The expression of each gene was calculated relative to the control sample at 20 CH.
